# Non-Cell-Autonomous Neurotoxicity in Parkinson’s Disease Mediated by Astroglial α-Synuclein

**DOI:** 10.1016/j.stemcr.2019.01.011

**Published:** 2019-02-12

**Authors:** MuhChyi Chai, Jun Kohyama

**Affiliations:** 1 Department of Physiology, Keio University School of Medicine, 35 Shinanomachi, Shinjuku-ku, Tokyo 160-8582, Japan; 2 Keio University Global Research Institute (KGRI), Keio University, 2-15-45, Mita, Minato-ku, Tokyo 108-8345, Japan

## Abstract

Non-cell-autonomous effects on neuronal cells are considered to be involved in the pathogenesis of neurodegenerative diseases but have yet to be mechanistically proven. In this issue of *Stem Cell Reports*, di Domenico et al. provide direct evidence that α-synuclein transferred from astrocytes exerts non-cell-autonomous neuronal dysfunction on dopaminergic neurons in Parkinson’s disease (PD).

## Main Text

Parkinson’s disease (PD) is the second most prevalent neurological disorder with varied pathological characteristics, including neuronal degeneration and gliosis in the substantia nigra pars compacta (SNpc), locus coeruleus, and dorsal motor nucleus of the vagal nerve (Dickson, 2018). A common phenomena of the disease progression is aggregates of disease-specific proteins. In PD, the deposition of Lewy bodies (LBs), which mainly consist of α-synuclein, is a pathological hallmark (Dickson, 2018). In addition to the accumulation of α-synuclein aggregates, neuron-to-neuron spreading of the aggregates through a “prion-like” mechanism has also been confirmed in various cellular and animal models (Dehay et al., 2016, Lee et al., 2012), such that the propagation of α-synuclein aggregates is correlated with clinical progression. Whereas the emphasis of PD-related research has been the relationship between α-synuclein and neuronal dysfunction, the possible involvement of astrocytes in the accumulation and propagation of α-synuclein has been overlooked.

**Figure 1:**
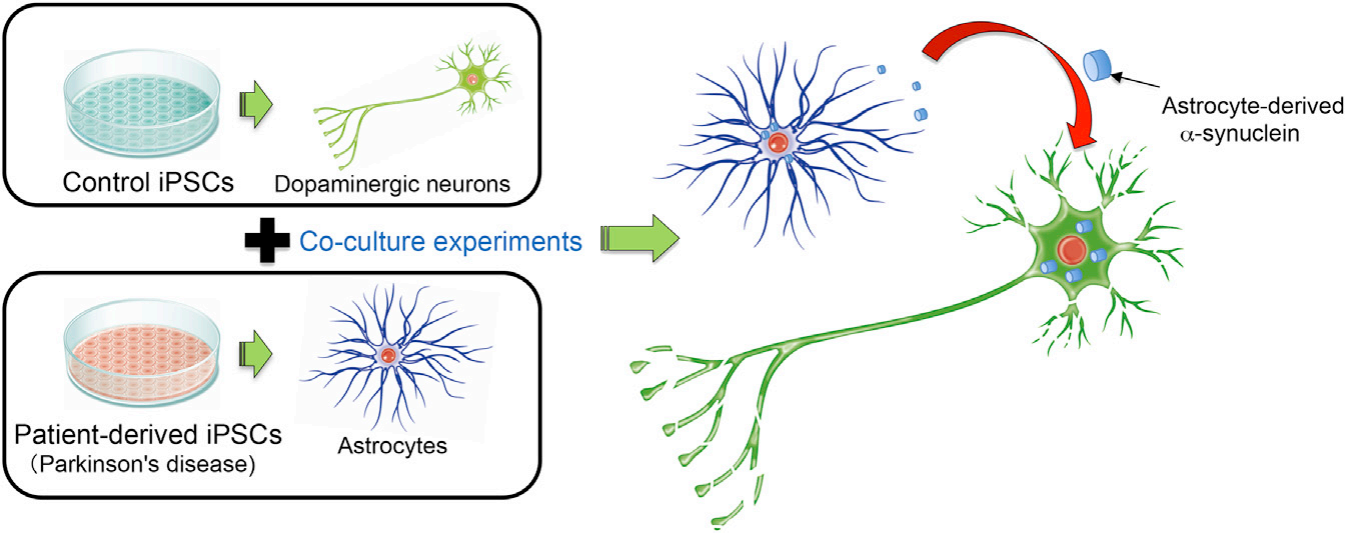
Astrocyte-Mediated Neurotoxicity in Parkinson’s Disease α-synuclein secreted from Parkinson’ disease patient-derived astrocytes exerts neurotoxic function on dopaminergic neurons, leading to non-cell-autonomous neuronal dysfunction in Parkinson’s disease.

In the CNS, astrocytes are the principal cell type that maintains homeostasis and confers neuroprotection, as facilitated by their close interaction with neurons. Astrocytic dysfunctions are causally associated with neuronal morphological and functional abnormalities that contribute to the progression of several neurodegenerative and neurodevelopmental diseases such as Alzheimer’s disease, amyotrophic lateral sclerosis, and Rett syndrome. In support of these findings, in this issue of *Stem Cell Reports*, di Domenico et al. (2019) describe an astrocyte-autonomous process mediating PD-associated degeneration of co-cultured iPSC-derived dopaminergic neurons, mainly via intracellular accumulation of α-synuclein aggregates in astrocytes and subsequent propagation of such toxic aggregates to surrounding neurons. As a result, human iPSC-derived dopaminergic neurons from healthy individuals perish in the presence of iPSC-derived astrocytes from PD patients with a *LRRK2*^G2019S^ mutation. Notably, the present study provides compelling evidence on how glia, in addition to neurons, contribute to the etiology of PD (Figure 1).

Mutations in the *LRRK2* locus are the most common cause of inherited PD, particularly the G2019S substitution mutation in *LRRK2*. Histological examinations of post-mortem brain tissues of PD patients with *LRRK2* mutations, including those with G2019S mutations, show neuronal loss in the SN, and nearly half of the post-mortem brain tissues exhibit α-synucleinopathy (Ross et al., 2006). Furthermore, findings gathered from *LRRK2*^G2019S^-mutated iPSC-derived dopaminergic neurons of PD patients and *Lrrk2* transgenic mice indicate that mutant LRRK2 toxicity in neurons was attributable to perturbations of autophagy and lysosomal pathways, including macroautophagy and chaperone-mediated autophagy (Orenstein et al., 2013). The impairment of these pathways interferes with protein degradation, which includes that of endogenous α-synuclein, thereby allowing the aggregation of α-synuclein. In line with this, chemical-induced clearance of α-synuclein accumulated in neuronal cultures markedly mitigates mutant LRRK2-mediated toxicity, substantiating a direct correlation between α-synuclein and LRRK2 mutations in PD-associated neurodegeneration (Orenstein et al., 2013). Of note, while such correlation has been abundantly demonstrated in neurons, besides dopaminergic neurons, abnormal deposition of α-synuclein was also visible in the astrocytes of post-mortem PD tissue. Nevertheless, the pathological consequences of mutant LRRK2 toxicity in astrocytes remain largely unexplored. di Domenico and colleagues found that the iPSC-derived astrocytes of PD patients are featured by extensive α-synuclein accumulation, including both monomeric and oligomeric α-synuclein, in which the latter constitutes the pathogenic form of α-synuclein. Within the cytoplasm of PD astrocytes, α-synuclein protein was persistently associated with LAMP2A-positive lysosomes, which otherwise will be degraded via the CMA pathway. Using a photoactivatable CMA reporter, the authors further confirmed that CMA activity was compromised in PD astrocytes. Although an alternative autophagy pathway such as macroautophagy is occasionally upregulated to compensate the reduced activity of CMA, this was not the case for PD astrocytes, which showed enhanced accumulation of autophagic vacuoles as a consequence of impaired autophagic flux. Based on these collective findings, the authors propose that the accumulation of α-synuclein aggregates in PD astrocytes is associated with impaired CMA and macroautophagy pathways. Surprisingly, the aberrant accumulation of α-synuclein aggregates did not affect the viability of PD astrocytes per se, but these astrocytes were found harmful to healthy dopaminergic neurons in a series of *in vitro* co-culture assays reported in the current study. The authors demonstrate that the interaction between *LRRK2*^G2019S^-bearing astrocytes and control neurons led to shorter, fewer, and dystrophic neurites of control neurons, as well as increased neuronal loss. This finding suggests that iPSC-derived astrocytes harboring *LRRK2*^G2019S^ mutations contribute to non-cell-autonomous neuronal damage/neurotoxicity of surrounding neurons.

Among the most interesting findings by di Domenico et al. (2019) is perhaps how PD astrocytes mediate mutant LRRK2 toxicity in iPSC-derived dopaminergic neurons. To show that α-synuclein-mediated neurotoxicity is triggered upon glia-to-neuron transmission of α-synuclein, the authors developed SNCA-flag tagged astrocyte lines using a CRISPR/Cas9-mediated knockin system and showed the unprecedented transfer of PD astrocyte-derived α-synuclein to surrounding control neurons in their cell-based models. In particular, these findings provide important insights into cellular transmission of pathological α-synuclein, which was previously only shown for neuron-to-neuron and neuron-to-glia transmission, and not vice versa. However, the underlying mechanisms facilitating the transmission of pathogenic α-synuclein from PD astrocytes to neurons remain unaddressed in the present study. In contrast to PD astrocytes, control astrocytes, when co-cultured with LRRK2-mutated iPSC-derived dopaminergic neurons, exerted neuroprotective effects. The control astrocytes internalized neuronal α-synuclein, thereby contributing to the clearance of α-synuclein accumulation and improving neuronal survival. di Domenico et al. (2019) also examined whether the addition of a CMA activator compound, QX77.1, to their cell-based models (PD astrocytes co-cultured with control or PD neurons) could restore the degradation of α-synuclein and subsequently inhibit PD astrocyte-mediated neurodegeneration. Following the treatment, they found that CMA was reactivated in PD astrocytes, as evidenced by restored perinuclear distribution of LAMP2A-positive lysosomes and decreased α-synuclein accumulation. Despite the clearance of α-synuclein in both the PD astrocytes and the surrounding control neurons, neuronal loss was only partially inhibited, suggesting that α-synuclein accumulation caused by defective CMA is unlikely the only “poisoning” feature of PD astrocytes.

While di Domenico et al. (2019) provide direct evidence of how astrocyte dysfunction can lead to PD-associated neurodegeneration, some questions still remain to be answered. For example, the current study does not examine oxidative stress, mitochondrial protein transport, and inflammatory responses in the corresponding PD astrocytes, considering the established role of LRRK2 in these cellular pathways (Nguyen et al., 2011). In addition, astrocytes in different brain regions possess functional heterogeneity, as exemplified by the findings that ventral, but not dorsal, astrocytes in the spinal cord are specifically important for the maintenance and survival of motor neurons (Molofsky et al., 2014). While the present study reported the successful generation of highly pure and functional human iPSC-derived astrocytes, the molecular identities of these astrocytes are unknown. In particular, further experiments can be conducted to clarify whether or not these astrocytes possess the regional specific identity that is necessary for the support of midbrain dopaminergic neurons. Meanwhile, a recent study showed that upon aging, astrocyte-specific genes are the most susceptible to changes in region-specific gene expression, especially in the hippocampus and SN (Soreq et al., 2017). Considering that disease penetrance in *LRRK2*^G2019S^ carriers increases with age (80% at age 80 years), how these multiple factors (astrocyte-specific genes, region-specific gene expression, and aging) can be incorporated and manipulated in the current cellular model is of great interest. Furthermore, revealing the mechanisms underlying the glia-to-neuron transmission of α-synuclein warrants further exploration. Nevertheless, the future findings will undoubtedly contribute to profound insights into the complexity of PD.
